# The outcomes of integrating biological interactions into rebuilding plans depend on prey specialization

**DOI:** 10.1002/eap.70269

**Published:** 2026-06-05

**Authors:** Andrea N. Odell, Kiva L. Oken, Marissa L. Baskett

**Affiliations:** ^1^ Department of Environmental Science and Policy University of California Davis California USA; ^2^ Fishery Resource Analysis and Monitoring Division, Northwest Fisheries Science Center National Marine Fisheries Service, National Oceanic and Atmospheric Administration Seattle Washington USA

**Keywords:** age‐structured models, ecosystem‐based fisheries management, groundfish, *Ophiodon elongatus*, recovery, *Sebastes ruberrimus*

## Abstract

Ecosystem dynamics can lead to trade‐offs between reaching harvest targets and protecting vulnerable species across fisheries management decisions. However, in the context of rebuilding overfished populations, considering predator–prey interactions might provide opportunities to minimize or reverse these trade‐offs if overfished prey can recover when predators in shared habitat are harvested. To understand whether and under what conditions such opportunities might arise, we explore the effect of predator harvest on the rebuilding outcomes of a recovering prey that experiences bycatch mortality. We developed an age‐structured model with predation and harvest to evaluate changes in the population dynamics of prey at steady state and in their rebuilding time under increasing harvest of predators. We parameterized our models based on yelloweye rockfish (*Sebastes ruberrimus*), a U.S. West Coast Groundfish stock under a rebuilding plan, and one of their known predators, lingcod (*Ophiodon elongatus*). We found that lingcod harvest reduced the long‐term spawning biomass and increased the rebuilding time of yelloweye rockfish regardless of their prey specialization; these negative effects were due to yelloweye rockfish bycatch in the lingcod fishery. However, the degree to which predator harvest affects prey rebuilding depends on prey specialization, where the steady‐state dynamics of yelloweye rockfish were less affected by lingcod harvest and rebuilding occurred more rapidly when lingcod acted as a specialist compared to a generalist predator. As efforts to leverage ecosystem attributes in fisheries management are applied to recovery strategies, we highlight the role that the nature and strength of biological interactions can play in shaping outcomes of recovery.

## INTRODUCTION

Recovery of overfished species occurs within ecosystems where multiple species coexist, interact, and are harvested simultaneously. Species differ in their capacity to withstand fishing pressure due to variation in productivity and life history traits (Beverton & Holt, 1957; Hilborn & Walters, 1992; Levin et al., 2006). Applying uniform fisheries management strategies across multiple species might impede recovery when less productive species are disproportionately impacted while constraining the sustainable harvest of more productive species. This trade‐off can be exacerbated when multiple species are captured simultaneously, whether purposefully in a mixed‐stock fishery or as bycatch (Burgess et al., 2013; Hilborn et al., 2012; Moore et al., 2021; Ricker, 1958). In such cases, outcomes of recovery likely depend on both fishing rates and how species interact with one another ecologically.

Biological interactions, such as predator–prey dynamics, can cause cascading and unintended outcomes from fisheries management strategies, whether for recovery planning or food production. For example, harvest of prey can reduce the productivity and harvest potential of predators through food limitation (Cury et al., 2011; May et al., 1979). Alternatively, harvest of predators can release prey from predation pressure, increasing the productivity of prey and bolstering their recovery. However, such positive outcomes for prey can also depend on the extent of fishing mortality they experience (e.g., bycatch) as it can outweigh the benefits of reduced predation pressure (Aalto & Baskett, 2013). Fishing mortality and predation pressure can also disrupt age structure and population stability (Planque et al., 2010), increasing uncertainty in population recovery (Hutchings & Reynolds, 2004; Shelton & Mangel, 2011). Therefore, considering how biological interactions might affect management decisions is a core component of ecosystem‐based fisheries management (Botsford et al., 1997; Lotze, 2004; Pikitch et al., 2004).

While many components of ecosystem‐based fisheries management have been well studied (Link & Marshak, 2022), incorporating biological interactions into decisions regarding the recovery of overfished species (e.g., rebuilding plans) is an emerging area of research (e.g., Dunn et al., 2021; Samhouri et al., 2017) compared to multispecies fisheries management. Rebuilding plans are individually implemented for overfished stocks to recover these populations to biomass levels that produce maximum sustainable yield (MSY). While rebuilding plans are a complex management approach, in this paper we focus primarily on the recovery measures typically implemented (e.g., reduced quota, spatial closures, fishery moratoria) which rapidly reduce the direct harvest and bycatch of rebuilding species (Murawski, 2010). Reducing fishing mortality is largely successful at restoring overfished species (Melnychuk et al., 2021) but can affect fishing opportunities for healthier populations within the fishery (Brodziak et al., 2004; Murawski, 2010). Strategically integrating biological interactions into strategies for recovery such as rebuilding plans can inform decisions to balance conservation goals with sustainable harvest opportunities.

Outcomes of recovery among interacting species depend on the interaction between harvest decisions and ecological processes (Baskett et al., 2006; Mangel & Levin, 2005; Marshall et al., 2016). The order of recovery between interacting species (e.g., simultaneous or sequential, where sequential entails continued harvest of one species while the other falls under protection) can affect how efficiently each of the interacting species can recover. For example, in a simple predator–prey system, simultaneous protection of both predator and prey yields the most efficient recovery for both species (Samhouri et al., 2017). When size structure is considered, however, sequential protection can increase recovery efficiency. In this case, predation that is limited to a subset of smaller prey sizes due to gape limitation reduces the likelihood of prey reaching reproductive sizes which can hinder their recovery (Dunn et al., 2021). We might expect that recovery efficiency in size‐structured populations can further depend on the degree of prey specialization. For example, gape‐limited specialist predation leads to greater fluctuations in the recovery of prey compared to gape‐limited generalist predation (Aalto & Baskett, 2017; Harvey et al., 2008). Size‐structured interactions and the degree of prey specialization are likely to interact and affect the efficiency of sequential or simultaneous recovery; understanding the outcomes of each can provide insight into the potential for an ecosystem‐based approach to recovery planning.

The US West Coast groundfish fishery (USWCGF) represents an ideal case study where the conditions of biological interactions, such as size structure and prey specialization, might affect outcomes of recovery for populations under a rebuilding plan. The fishery was declared a federal disaster in 2000 following years of intense fishing by an overcapitalized fleet leading to its collapse (Warlick et al., 2018). Ten species were declared overfished and fell under rebuilding plans which included simultaneous protection via expansive spatial closures between California and Washington called Rockfish Conservation Areas (RCAs; Warlick et al., 2018). Yelloweye rockfish (*Sebastes ruberrimus*) remained the only species under a rebuilding plan two decades later, which had raised concerns regarding the consequent underutilization of more productive stocks (PFMC, 2018). For example, lingcod (*Ophiodon elongatus*) is a productive species whose fishery has historically incurred rockfish as bycatch (Lowry et al., 2024). Lingcod are known to consume juvenile rockfish (Beaudreau & Essington, 2007; Brown, 2021; Tinus, 2012), and an equilibrium‐based model of their interaction suggests that rockfish could have greater biomass recovery if lingcod were harvested within the RCA with limited bycatch of rockfish, especially if lingcod are more specialized on rockfish (Oken & Essington, 2016). In addition to biomass recovery, however, recovery time is another important consideration in designing rebuilding plans. Further investigation into both the transient dynamics and the uncertainty associated with outcomes of recovery can provide a more holistic understanding of recovery strategies, such as rebuilding plans, that consider biological interactions.

In this paper, we quantify how prey specialization interacts with size‐structured predation and bycatch mortality to affect the outcomes of recovery for an overfished population under a rebuilding plan (e.g., yelloweye rockfish). We integrate aspects of our current theoretical understanding of recovery among interacting species, such as order of recovery (Samhouri et al., 2017) and population structure (Dunn et al., 2021), to explore alternative strategies of recovery that include predator harvest. We use yelloweye rockfish and their predator, lingcod, in the USWCGF as a relevant case study to ground our model. While, in this study, we focus primarily on the recovery dynamics of yelloweye rockfish, further research using this model can explore the implications of alternative recovery strategies (e.g., lingcod harvest) on the population dynamics and fisheries yield of lingcod.

To this end, we evaluate an age‐structured predator–prey model with size‐structured predation, fishery mortality, and stochastic recruitment to drive population variability. We explored two questions: (1) How does lingcod prey specialization affect yelloweye rockfish long‐term rebuilding outcomes (spawning biomass, stability, and age structure at steady state) in the absence of lingcod fishing? and (2) How does lingcod harvest affect both the long‐term and transient rebuilding outcomes (rebuilding time) of yelloweye rockfish under different prey specialization scenarios? We expect that the long‐term and transient outcomes of yelloweye rockfish under a rebuilding plan will improve with increased harvest of lingcod between low to moderate fishing pressure. Within this range of fishing pressure, we expect the positive effect of predator release to outweigh the negative effect of bycatch mortality.

## METHODS

### Study system

The USWCGF is composed of over 90 species with contrasting life histories including rockfish, roundfish, flatfish, and elasmobranchs (PFMC, 2022). Our focal species, lingcod and yelloweye rockfish, are demersal groundfish that occupy rocky reef habitats with overlapping distributions along the U.S. West Coast and were declared overfished in 1999 and 2002, respectively (Gertseva & Cope, 2017; Haltuch et al., 2018). Lingcod are a productive species that exhibit sexual dimorphism (Cass et al., 1990), and latitudinal differences in genetic and growth characteristics separate the population into northern and southern stocks for assessment purposes (Haltuch et al., 2018; Lam et al., 2021; Longo et al., 2020). Their productivity is driven by rapid growth and early maturation (50% maturity at age 2 for males and age 3–5 for females), and they can live up to approximately 22 years (Cass et al., 1990). In comparison, yelloweye rockfish are less productive due to their slower growth, late maturity (50% maturity at age 15–20 for females and males; Yamanaka & Kronlund, 1997), and longer lifespans (up to 150 years for females and males; Gertseva & Cope, 2017). As a result of these life history differences, lingcod have been declared rebuilt since 2005 while yelloweye rockfish has remained below the rebuilding plan's biomass target two decades after its establishment (PFMC, 2020). Yelloweye's rebuilding status and very low allowable quota has constrained the catch of other groundfish, such as lingcod, because they can co‐occur in catch by demersal fishing gear. While lingcod are generalist predators that consume many species, a dramatic increase in lingcod abundance following their recovery could result in greater predation pressure on yelloweye rockfish, potentially hindering their recovery. Both species are iteroparous with infrequent large recruitment events linked to environmental variability (Charapata & Trumble, 2023; Stachura et al., 2014), which can also influence recovery dynamics.

### Model overview

**FIGURE 1:**
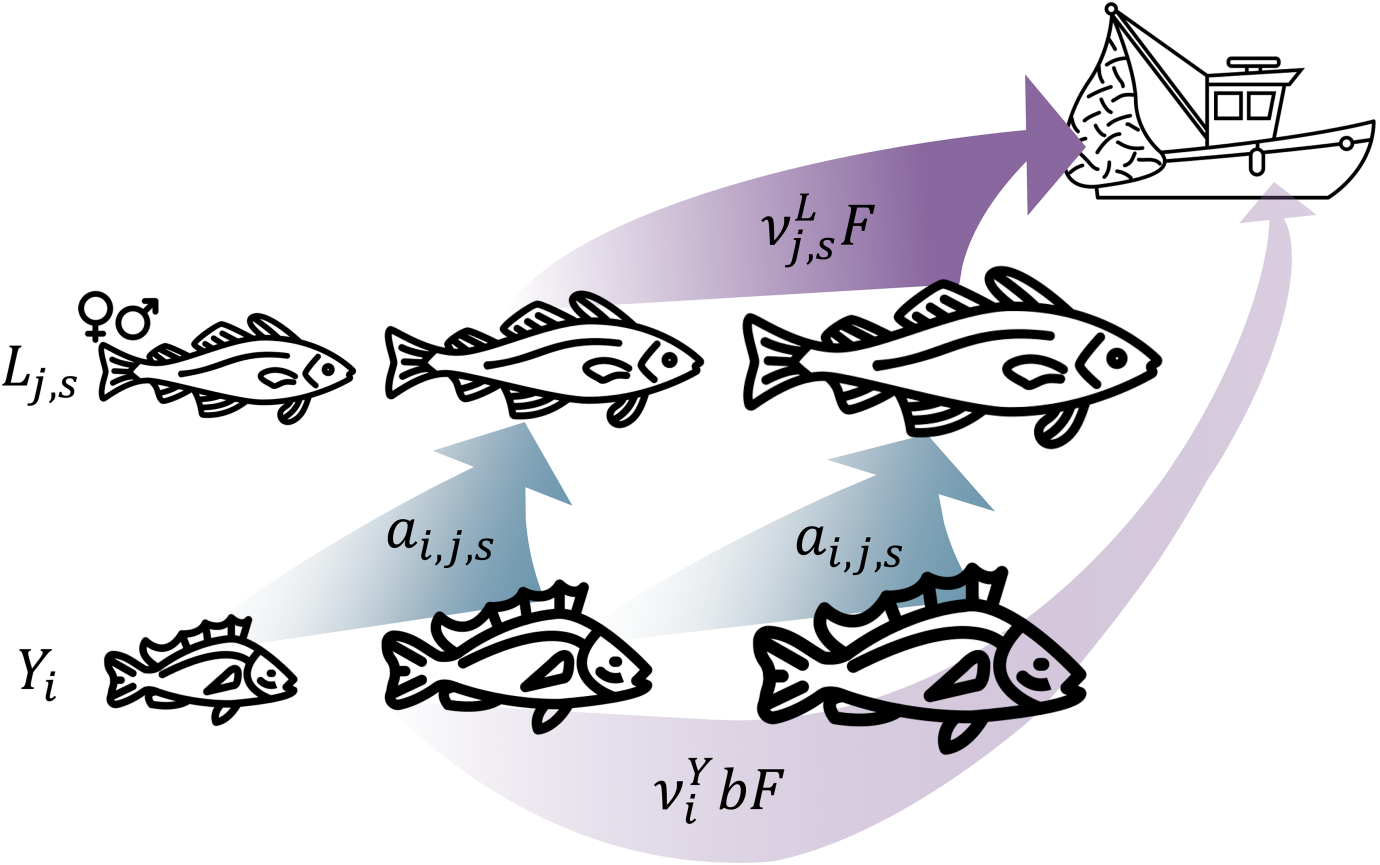
Conceptual model of yelloweye at age *i* ($Y_{i}$) and lingcod at age *j* and sex *s* ($L_{j,s}$), where the arrows represent mortality through either consumption (blue) or fishing (purple). Lingcod selectively consume yelloweye by size ($a_{i,j,s}$). A fishery directly harvests vulnerable sizes of lingcod ($v_{j,s}^{L}F$) and incidentally captures yelloweye proportional to targeted fishing pressure ($v_{i}^{Y}\mathrm{bF}$). Model schematic was adapted from (Oken & Essington, 2016). Icons were obtained from Noun Project CC BY 3.0 and created by Laymik (“cod”), Iyikon (“rockfish”), and Martin Lebreton (“fishing boat”).

We modeled the age‐structured dynamics of yelloweye rockfish (hereafter referred to as yelloweye) and lingcod, where we converted age to size to represent size‐dependent predation (Figure 1). We modeled sex explicitly for lingcod given their sexual dimorphism. We aggregated yelloweye across sex as sexual dimorphism is not observed (Gertseva & Cope, 2017). For simplicity, we assumed that all dynamics occurred within the RCA and are thus considered closed populations. We assumed that lingcod productivity does not depend on their consumption of yelloweye because they are opportunistic generalists. We assumed that lingcod consumption of yelloweye (and other prey species) was dependent on both prey density because of the time needed to process food (Holling, 1959) and on size because of gape limitation (Beaudreau & Essington, 2007; Frid et al., 2013). We included stochastic, density‐dependent recruitment for both species, where the recruitment deviations were autoregressive. We simulated the numbers‐at‐age of yelloweye and lingcod through time to analyze the (1) long‐term outcomes of yelloweye measured as the spawning biomass, stability, and age structure at steady‐state and (2) transient rebuilding outcomes measured as rebuilding time.

### Model structure

We used a semi‐discrete model (Mailleret & Lemesle, 2009) that follows natural, fishing, and, for yelloweye only, predation mortality over continuous time between discrete, annual pulses of recruitment at time $\tau_{k}$, for *k* = 1, …, $T_{\mathrm{Max}}$. We tracked the number of age j and sex s lingcod individuals, $L_{j,s}$, over time. Lingcod experience sex‐specific natural mortality at a rate $M_{s}^{L}$ and fishing mortality at a rate F. Because fishing behavior (e.g., mesh sizes, area, and size restrictions) impacts the sizes of lingcod that tend to be captured, vulnerability to fishing $\nu_{j,s}^{L}$ modified the fishing mortality rate. For simplicity, we set $\nu_{j,s}^{L}$ to either 0 (not vulnerable) or 1 (fully vulnerable). We specified a final age class, J, after which mortality and reproductive output remain the same due to growth saturation, such that J represents all individuals older than the second to last age class (known as a “plus group”). The change in $L_{j,s}$, where j=1,…,20, over time t is then

(1) $$\frac{d}{\mathrm{dt}}L_{j,s}=-\left(M_{s}^{L}+\nu_{j,s}^{L}F\right)L_{j,s}\;\text{for}\;t\neq \tau_{k}.$$

Advancement from age j to j+1 occurs in discrete annual pulses, indexed by $\tau_{k}^{+}$, the instant following natural and fishing mortality. We assumed discrete annual pulses of density‐dependent recruitment that follow a saturating Beverton–Holt function defined by parameters $\beta^{L}$ and $\alpha^{L}$ which describe the strength of density dependence and the production per total spawning biomass, respectively. We calculated spawning biomass $S^{L}$ for each time step by converting the total number of sexually mature female lingcod to biomass and summed biomass across all age classes. Specifically, $S^{L}=\sum_{j}L_{j,F}W_{j,F}m_{j,F}$, where $W_{j,F}$ is a vector of weight‐at‐age for female lingcod and $m_{j,F}$ is a vector of the proportion of mature females in each age class. We incorporate stochastic recruitment using an autoregressive time series of exponentiated random normal deviations, $\epsilon^{L}$, with mean zero, strength of autocorrelation $\rho_{L}$, and SD σ. The number of age‐1 lingcod added to the population at time $\tau_{k}^{+}$, $L_{1,s}\left(\tau_{k}^{+}\right)$, is therefore

(2) $$L_{1,s}\left(\tau_{k}^{+}\right)=\frac{0.5\alpha^{L}S^{L}\left(\tau_{k}\right)}{1+\beta^{L}S^{L}\left(\tau_{k}\right)}e^{\epsilon_{\tau_{k}^{+}}^{L}-0.5\sigma^{2}},\text{where}\;\epsilon_{\tau_{k}^{+}}^{L}|\epsilon_{\tau_{k-1}^{+}}^{L}\sim \text{Normal}\left(\rho_{L}\epsilon_{\tau_{k-1}^{+}}^{L},\sigma^{2}\left(1-\rho_{L}^{2}\right)\right).$$

Because we modeled sex explicitly for lingcod, 0.5 in the numerator accounts for the sex ratio at birth. The term $-0.5\sigma^{2}$ in the exponent is a bias correction factor to ensure that the expectation of the exponentiated term is one.

We derived the parameters α^*L*^ and β^*L*^ from the age‐structured model using species‐specific information on the steepness of the slope of the stock‐recruitment relationship *h* ^*L*^, recruitment at carrying capacity $R_{0}^{L}$, and recruits per spawner at carrying capacity ϕ^*L*^ following the forms $\alpha^{L}=\frac{4h^{L}}{\phi^{L}\left(1-h^{L}\right)}$ and $\beta =\frac{5h^{L}-1}{\phi^{L}R_{0}^{L}\left(1-h^{L}\right)}$ (Table 1). A $h^{L}$ of one indicates constant recruitment independent of spawning biomass and a $h^{L}$ of 0.2 indicates a linear relationship between recruitment and spawning biomass. All post‐recruitment age classes 1≤j<J advance in age according to $L_{j+1,s}\left(\tau_{k}^{+}\right)=L_{j,s}\left(\tau_{k}\right)$ and, for the plus group of individuals age J =20 and older, $L_{J,s}\left(\tau_{k}^{+}\right)=L_{J,s}\left(\tau_{k}\right)+L_{J-1,s}\left(\tau_{k}\right)$.

**TABLE 1:** Summary of model parameters.

Symbol	Description	Value	References
Lingcod			
$M_{m}^{L}$	Natural mortality rate—male	0.32 year^−1^	Haltuch et al. (2018)
$M_{f}^{L}$	Natural mortality rate—female	0.18 year^−1^	Haltuch et al. (2018)
$\nu_{j,s}^{L}$	Age *j* sex *s* vulnerability to fishing	0 or 1	
*h* ^*L*^	Beverton–Holt steepness	0.8	Haltuch et al. (2018)
$R_{0}^{L}$	Recruitment at carrying capacity	4848	Haltuch et al. (2018)
$k_{m}^{L}$	von Bertalanffy growth coefficient—male	0.214 year^−1^	Haltuch et al. (2018)
$k_{f}^{L}$	von Bertalanffy growth coefficient—female	0.191 year^−1^	Haltuch et al. (2018)
$L_{\min}^{L}$	Minimum length lingcod caught by fishery	56 cm	Haltuch et al. (2018)
$a_{m}^{c}$	Lingcod consumption intercept—males	3.01	Beaudreau and Essington (2009)
$a_{f}^{c}$	Lingcod consumption intercept—females	3.31	Beaudreau and Essington (2009)
$b_{m}^{c}$	Lingcod consumption exponent—males	0.77	Beaudreau and Essington (2009)
$b_{f}^{c}$	Lingcod consumption exponent—males	0.71	Beaudreau and Essington (2009)
*Y*_*5*_	Slope of fifth quantize of diet size spectra	0.05	Beaudreau and Essington (2007)
*Y*_*95*_	*Slope of ninety‐fifth quantize of diet size spectra	0.29	Beaudreau and Essington (2007)
$L_{m}^{\infty }$	Maximum length—male	86.3 cm	Haltuch et al. (2018)
$L_{f}^{\infty }$	Maximum length—female	100.9 cm	Haltuch et al. (2018)
$a_{m}^{w}$	Mass‐length intercept—male	2.179E‐6	Haltuch et al. (2018)
$a_{f}^{w}$	Mass‐length intercept—female	3.308E‐6	Haltuch et al. (2018)
$b_{m}^{w}$	Mass‐length intercept—male	3.36	Haltuch et al. (2018)
$b_{f}^{w}$	Mass‐length intercept—female	3.248	Haltuch et al. (2018)
$\rho_{L}$	*Recruitment autocorrelation	0.23	Black et al. (2008)
σ	*Recruitment c.v.	0.5	Haltuch et al. (2018)
δ	*Handling time	0.3	
$\phi^{L}$	Recruits per spawner at carrying capacity		Derived from age‐structured model
Yelloweye			
*M* ^*Y*^	Natural mortality rate	0.044 year^−1^	Gertseva and Cope (2017)
*h* ^*Y*^	Beverton–Holt steepness	0.718	Gertseva and Cope (2017)
$\nu_{i}^{Y}$	Age *i* vulnerability to fishing	0 or 1	
$a_{i,j,s}$	Matrix of size‐selective rate of consumption		See Appendix S1
*q*	Alternate prey availability		See Appendix S1
$R_{0}^{Y}$	Recruitment at carrying capacity	220	Gertseva and Cope (2017)
*k* ^*Y*^	von Bertalanffy growth coefficient	0.049 year^−1^	Gertseva and Cope (2017)
$L_{\min}^{Y}$	Minimum length caught by fishery	25 cm	
$L_{\infty }^{Y}$	Maximum length	63.9 cm	Gertseva and Cope (2017)
$a_{w}^{Y}$	Mass‐length intercept	7.313E‐6	Gertseva and Cope (2017)
$b_{w}^{Y}$	Mass‐length intercept	3.242	Gertseva and Cope (2017)
$\rho_{Y}$	*Recruitment autocorrelation	0.23	Black et al. (2008)
σ	*Recruitment c.v.	0.5	Gertseva and Cope (2017)
*b*	Bycatch mortality	0.05	
$\phi^{Y}$	Recruits per spawner at carrying capacity		Derived from age‐structured model

*Note*: Parameters indicated by an asterisk (*) were evaluated in the global sensitivity analysis.

We follow the number of age i yelloweye individuals, represented as $Y_{i}$ where i=1,…,65, as they continuously undergo dynamics of natural, fishing, and predation mortality between discrete annual pulses of recruitment. Natural and fishing mortality operate similarly to lingcod. Yelloweye experience baseline natural mortality at rate $M^{Y}$ that is fixed across all scenarios and bycatch mortality b proportional to the rate of fishing F experienced by lingcod. Because fisheries rarely target and capture small individuals, we also modified bF using age‐specific vulnerability to the fishery $\nu_{i}^{Y}$ set to either 0 or 1. For the predation mortality rate, which varies across prey specialization scenarios, we assumed lingcod consume yelloweye following a saturating Hollings Type 2 functional response with handling time δ, and alternate prey availability q, where the availability and handling time of alternative prey slows predation on yelloweye. To account for gape limitation, we specified a size‐selective rate of per capita consumption dependent on the combination of age *i* yelloweye and age j and sex s lingcod, represented as $a_{i,j,s}$. To calculate $a_{i,j,s}$, we combined three pieces of information: the total annual consumption by an age j and sex s lingcod, an age j and sex *s* lingcod's preference for different prey sizes, and the proportion of consumed biomass that is yelloweye specifically (which controls prey specialization; γ). We then converted these annual proportions to instantaneous rates. See Appendix S1: Section S1 for details on these calculations. The change in $Y_{i}$ over time between recruitment pulses is then

(3) $$\frac{d}{\mathrm{dt}}Y_{i}=-\left(M^{Y}+\nu_{i}^{Y}\mathrm{bF}\right)Y_{i}-\sum_{s}\sum_{j}\frac{a_{i,j,s}Y_{i}L_{j,s}}{1+\delta \sum_{i}\left(a_{i,j,s}Y_{i}\right)+\delta q}\;\text{for}\;t\neq \tau_{k}.$$

Yelloweye undergo annual pulses of recruitment and advancement from age *i* to age i+1 at time $\tau_{k}^{+}$. Recruitment followed a saturating Beverton–Holt function with a strength of density‐dependence $\beta^{Y}$ and production per total spawning biomass $\alpha^{Y}$. We incorporated recruitment stochasticity using an autoregressive time series of exponentiated random normal deviations, $\epsilon^{Y}$, with mean zero, strength of autocorrelation $\rho_{Y}$, and SD σ. The number of age‐1 recruits added to the population at time $\tau_{k}^{+}$, $Y_{1}\left(\tau_{k}^{+}\right)$, is then

(4) $$Y_{1}\left(\tau_{k}^{+}\right)=\frac{\alpha^{Y}S^{Y}\left(\tau_{k}\right)}{1+\beta^{Y}S^{Y}\left(\tau_{k}\right)}e^{\epsilon_{\tau_{k}^{+}}^{Y}-0.5\sigma^{2}},\text{where}\;\epsilon_{\tau_{k}^{+}}^{Y}|\epsilon_{\tau_{k-1}^{+}}^{Y}\sim \text{Normal}\left(\rho_{Y}\epsilon_{\tau_{k-1}^{+}}^{L},\sigma^{2}\left(1-\rho_{Y}^{2}\right)\right).$$

We derived the $\alpha^{Y}$ and $\beta^{Y}$ parameters following the same calculations used for lingcod. Post‐recruitment age classes 1≤i<I advance in age according to $Y_{i+1}\left(\tau_{k}^{+}\right)=Y_{i}\left(\tau_{k}\right)$ until they reach the plus group of individuals aged I=65 and older, where $Y_{I}\left(\tau_{k}^{+}\right)=Y_{I}\left(\tau_{k}\right)+Y_{I-1}\left(\tau_{k}\right)$.

We constructed and simulated all of our models in R v4.2 (R Core Team, 2023).

### Model analysis

Our model loosely represents our focal species, yelloweye and lingcod (south of 40°10′ N), to provide a foundation for the model from which we gain more general insights. We parameterized our model using information from statistical age‐structured stock assessments conducted in 2017 (Gertseva & Cope, 2017; Haltuch et al., 2018) and peer‐reviewed publications (Table 1). However, there is limited information for several parameters regarding the consumption of yelloweye rockfish by lingcod (indicated by an asterisk in Table 1), for which we used reasonable estimates and evaluated their influence using a global sensitivity analysis (GSA; Harper et al., 2011). In the GSA, we omitted fishing mortality (*F* = 0 across all simulations) and focused on the importance of key parameters associated with predation and recruitment. We ran 2000 Monte Carlo simulations that randomly sampled values from a uniform distribution unique to each parameter, providing 2000 unique parameterizations to simulate. Using a random forest algorithm from the *randomForest* package (v4.7‐1.1; Liaw & Wiener, 2002), we identified parameters associated with the strongest influence on the long‐term outcomes of yelloweye measured by the percent increase in mean squared error (MSE). Because the GSA simultaneously samples all selected parameters at once, this analysis captures interactions between parameters. See Appendix S1: Table S1 for parameter distributions.

**FIGURE 2:**
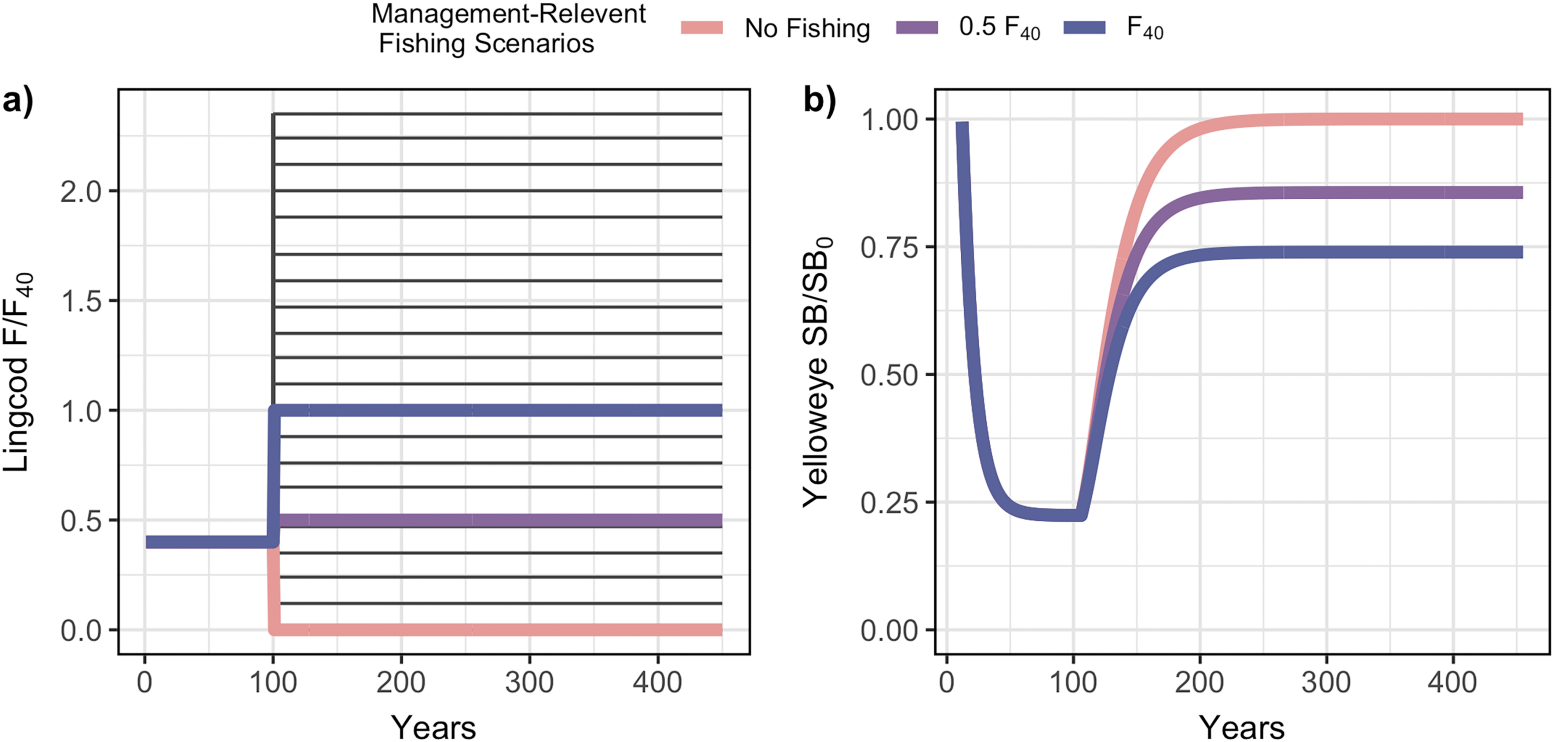
Timeseries of (a) all analyzed lingcod harvest scenarios (in dark gray lines) measured as the lingcod fishing rate relative to the rate that would achieve 40% unfished spawning biomass of lingcod at steady state ($\frac{F}{F_{40}}$) with three management‐relevant fishing scenarios highlighted (in color) and (b) the deterministic simulations of yelloweye spawning biomass (SB) relative to its unfished spawning biomass (SB_0_) for the three management‐relevant lingcod harvest scenarios. The colors indicate the management‐relevant scenarios of lingcod harvest analyzed: no fishing (*F* = 0), fishing at half the management target (*F* = 0.5*F*_40_), and fishing at management target (*F* = *F*_40_). We define the fishing rate at management target as the rate that results in lingcod exhibiting 40% of its unfished spawning biomass at steady state.

For all model analyses, we numerically simulated our model through time using the *lsoda* algorithm for ordinary differential equation integration in R v4.2 (R Core Team, 2023). We explored several combinations of prey specialization and lingcod harvest scenarios (Table 2). For each scenario, we ran 150 simulations for $T_{\mathrm{Max}}=450$ years which we determined to be an adequate length of time to reach a steady‐state distribution under stochastic recruitment (Figure 2). For each simulation, both populations started with high biomass across all ages; preliminary simulations confirmed that steady‐state outcomes were robust to changes in these initial starting conditions. We then deterministically simulated historical fishing pressure on lingcod and yelloweye for 100 years until both populations reached an exploited steady state that was 22% of their unfished biomass for each scenario. Once both populations reached their exploited steady state, we simulated a lingcod harvest scenario during rebuilding for the remaining 350 years where we varied *F* across scenarios (Figure 2). For each scenario, we quantified both the long‐term and transient rebuilding outcomes of yelloweye. We measured the long‐term outcomes as the mean spawning biomass, the inverse of the mean coefficient of variation of spawning biomass (stability; $CV^{-1}$), and the mean proportion of the total population within the plus group (age structure) over the last 150 years at steady state. We measured the transient rebuilding outcome as rebuilding time, that is, the number of years a yelloweye simulation took to reach 40% of its unfished spawning biomass, $0.4S_{0}^{Y}$. We selected $0.4S_{0}^{Y}$ as a relative biomass benchmark, and while 40% of unfished biomass is a common single‐species reference point (referred to as B40%), its main role here is to serve as a value to compare recovery time across scenarios. For each lingcod harvest scenario, we calculated rebuilding time and its uncertainty as the mean and SD across simulations.

**TABLE 2:** Combination of lingcod harvest and prey specialization scenarios.

Scenario	Prey specialization (γ)	Lingcod harvest (*F*)
Generalist	0.001	0, …, 0.4
Specialist	0.05	0, …, 0.4
None	0	0
Intermediate	0.014	0

*Note*: For generalist and specialist scenarios, we explored lingcod harvest scenarios ranging between *F* = 0 and *F* = 0.4. We did not explore fishing scenarios for no predation (“none”) and intermediate prey specialization scenarios.

To investigate the effect of lingcod prey specialization on the long‐term outcomes of yelloweye, we simulated our model without fishing mortality ($F=0\;\mathrm{yea}r^{-1}$) during the 350 years of rebuilding and focused primarily on the unfished ecological dynamics of this system. We fixed all parameters except for prey specialization γ, which is the proportion of yelloweye in lingcod diet and is a component of $a_{i,j,s}$ (see Appendix S1: Section S1). Given both the influence of and the degree of uncertainty in lingcod consumption of yelloweye, we tested a range of prey specialization within and beyond what we might expect between lingcod and yelloweye. In doing so, we hoped to gain insight into the relative importance of prey specialization between systems consistent with lingcod and rockfish as well as other more‐tightly coupled systems. Specifically, we explored four prey specialization scenarios: no interaction (γ=0), lingcod as a generalist predator informed by a diet study (γ=0.001; Beaudreau & Essington, 2009), lingcod as a specialist predator on yelloweye (γ=0.05), and lingcod as an intermediate predator between a generalist and specialist (γ=0.014). We based γ on a combination of two parameters: the proportion of rockfish in the diet of lingcod and the proportion of consumed rockfish that was yelloweye specifically. We later combined these parameters into γ for simplicity which ultimately led to a combined value for intermediate predators that was not equidistant from the values for generalist and specialist predators. While the specific outcomes might differ slightly, we expect the trends and overall conclusions to be similar. Across prey specialization scenarios, we evaluated differences in the spawning biomass, stability, and age structure of yelloweye at steady state.

To investigate the effect of lingcod harvest on the long‐term and transient rebuilding outcomes of yelloweye, we simulated the model with increasing fishing mortality during the 350 years of rebuilding (e.g., Figure 2a). We explored the effect of lingcod harvest for two prey specialization scenarios, generalist (γ=0.001) and specialist (γ=0.05), with a continuous range of *F* during yelloweye rebuilding that ranged from no fishing ($F=0\;\mathrm{yea}r^{-1}$) to fishing at an instantaneous rate of 40% of the fully selected sizes of the lingcod population ($F=0.4\;\mathrm{yea}r^{-1}$; Table 2). For each small increment in *F*, we ran 150 simulations. For all lingcod harvest scenarios, we assumed yelloweye experience low bycatch mortality (b=0.05; i.e., 5% of the fishing rate *F* on lingcod) and conducted a sensitivity analysis by doubling the bycatch parameter (b=0.1). Relative exploitation rates (catch/biomass of age 8+ fish) of yelloweye were estimated to be below 1% of the total population between 2007 and 2016 when capture of yelloweye was kept at a minimum (Gertseva & Cope, 2017). Therefore, we explored values of yelloweye bycatch mortality (bF in Equation 3) similar to the observed relative exploitation rates in addition to those likely to result from opening spatially protected areas to lingcod fishing. We calculated rebuilding time for three distinct lingcod harvest scenarios: no fishing, fishing at 50% of the management harvest target for lingcod (0.5*F*_40_), and fishing at the management harvest target for lingcod (*F*_40_; Figure 2a), where we defined the management harvest target (*F*_40_) as the fishing rate (*F*) that results in lingcod's spawning biomass at steady state to be equal to 40% of its unfished biomass.

## RESULTS

### Role of lingcod prey specialization on yelloweye rockfish dynamics

**FIGURE 3:**
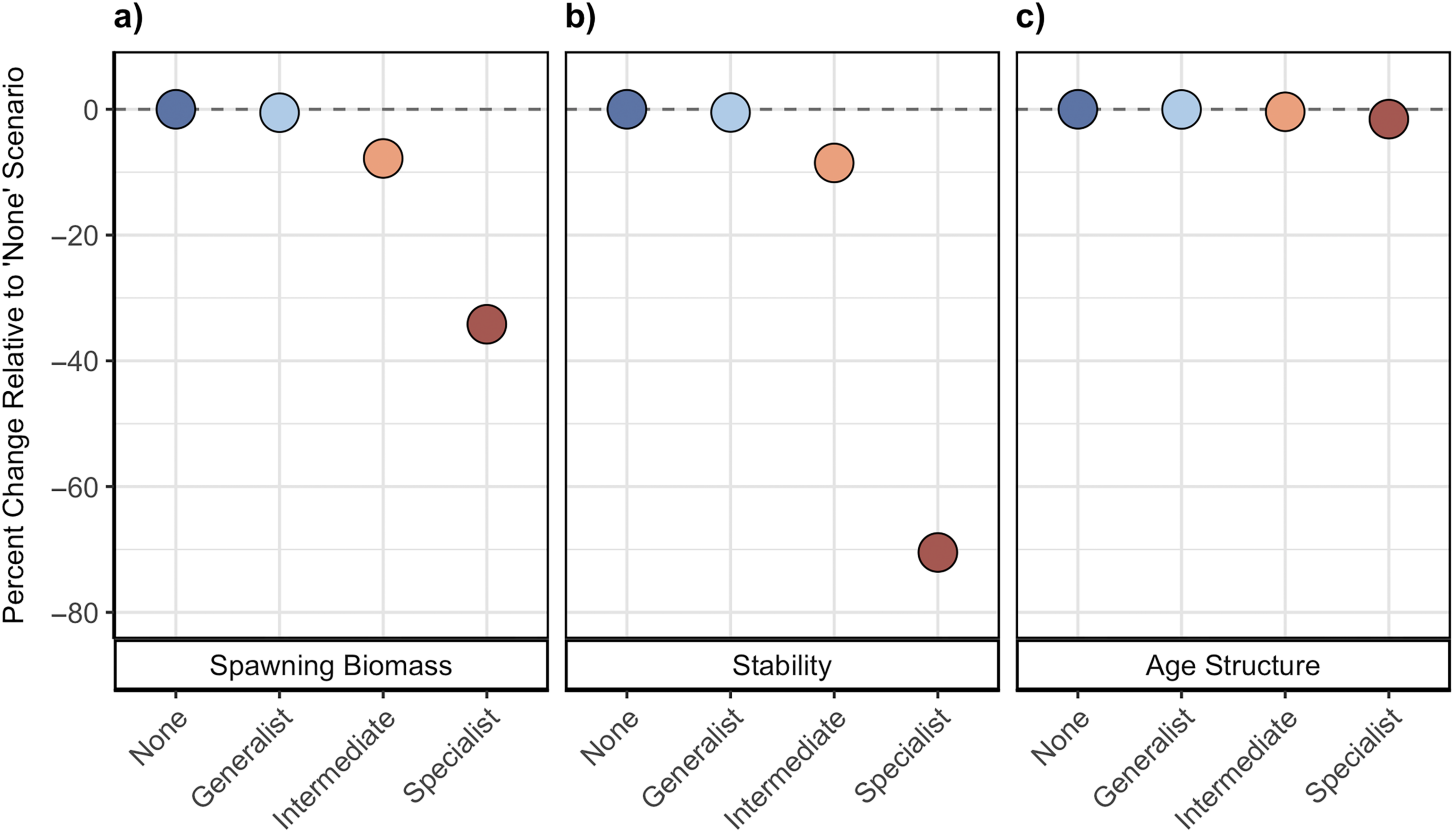
Steady‐state outcomes of yelloweye rockfish mean (a) spawning biomass, (b) stability, and (c) age structure over the last 150 years across simulations related to varying levels of prey specialization by lingcod: none (γ = 0), generalist (γ = 0.001), intermediate (γ = 0.014), and specialist (γ = 0.05). Fishing was set to F=0 across all prey specialization scenarios. We visualize outcomes in relation to the no biological interaction (“none”) scenario, where the values are represented as the precent change in outcome relative to the outcome if lingcod do not consume yelloweye rockfish.

In our exploration of different prey specialization scenarios without fishing, increased prey specialization by lingcod reduced the long‐term spawning biomass and stability of yelloweye, with little impact to age structure (Figure 3). When modeled as a generalist predator (γ=0.001; “Generalist” in Figure 3), considering predation by lingcod resulted in little change to the long‐term outcomes of yelloweye relative to when there was no predation (γ=0; “None” in Figure 3). However, when lingcod acted as a specialist predator (γ=0.05; “Specialist” in Figure 3), the long‐term yelloweye spawning biomass and stability reduced by 34% and 70%, respectively (Figure 3a,b). The level of yelloweye spawning biomass relative to other prey specialization scenarios remained consistent throughout recovery, indicating that transient dynamics follow similar patterns to steady‐state outcomes (Appendix S1: Figure S1). Reduced spawning biomass of yelloweye is a direct result of the increase in their consumption by lingcod at higher prey specialization. Meanwhile, reduced stability is a response to the stronger trophic coupling between yelloweye, a relatively stable prey, and lingcod, a highly dynamic and variable predator, causing yelloweye to more closely track variability in lingcod through this trophic pathway.

**FIGURE 4:**
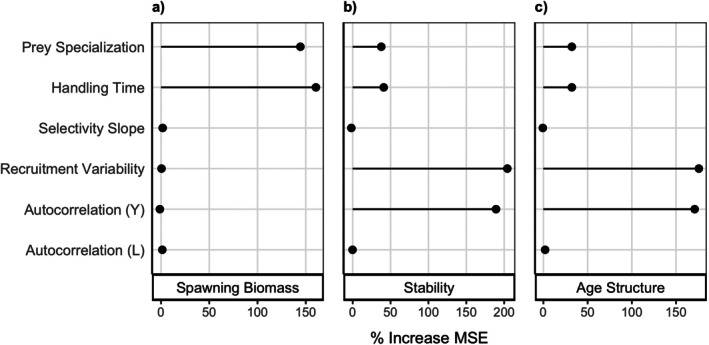
Outcomes of a global sensitivity analysis displayed using % increase in mean squared error (MSE) as a measure of importance for (a) spawning biomass, (b) stability, and (c) age structure. Increasingly positive values indicate greater influence (or importance) on model outputs and negative values occur when a random variable is comparably more predictive.

Results from our GSA revealed that the nature of lingcod predation played a primary role in determining yelloweye spawning biomass (Figure 4a), while recruitment dynamics influenced primarily stability and age structure (Figure 4b,c). Specifically, the parameters that most influenced spawning biomass of yelloweye at steady state were prey specialization (γ) and handling time (δ), while those that most affected stability and age structure were recruitment variability (σ) and yelloweye recruitment autocorrelation (ρ^*Y*^), with prey specialization (γ) and handling time (δ) showing intermediate influence (Figure 4). Therefore, while prey specialization had a dramatic effect on stability in the default parameterization (Figure 3b), any small change in recruitment variability or autocorrelation might have a larger effect as demonstrated by the GSA (Figure 4b).

### Effects of lingcod harvest on rebuilding outcomes of yelloweye rockfish

**FIGURE 5:**
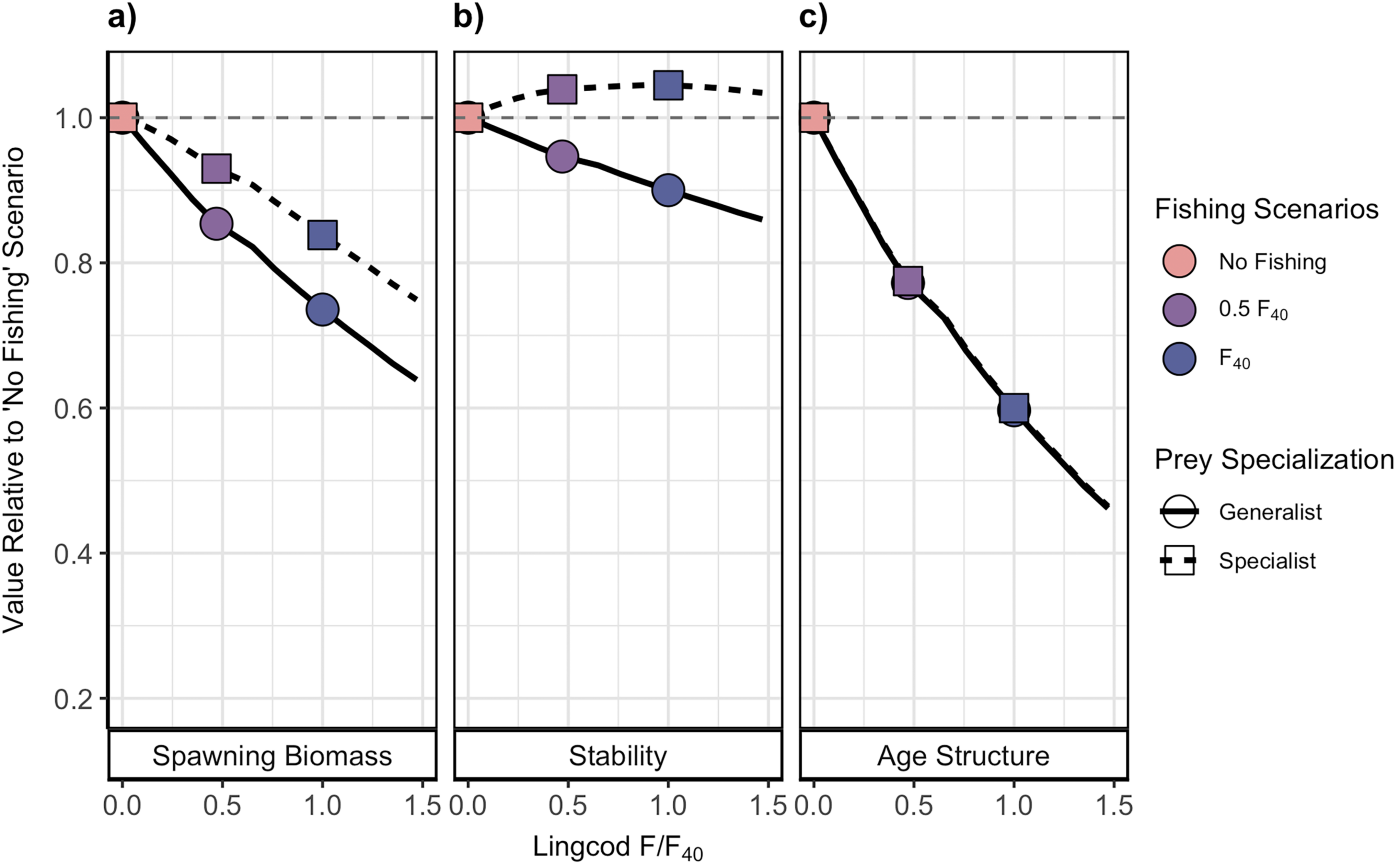
Steady‐state outcomes of yelloweye rockfish (a) spawning biomass, (b) stability, and (c) age structure related to increasing lingcod fishing pressure *F* relative to a fishing rate that achieves 40% of the unfished biomass for lingcod *F*_40_ for lingcod as a generalist predator (solid line with circles) and a specialist predator (dashed line with squares). We visualize outcomes in relation to the no fishing scenario ($\frac{F}{F_{40}}=0$) scenario, where the values are represented as the proportion relative to the outcome expected if a lingcod fishery did not operate. Colored shapes indicate three fishing scenarios ($\frac{F}{F_{40}}=0$, $\frac{F}{F_{40}}=0.5$, and $\frac{F}{F_{40}}=1$) explored for rebuilding time.

In our investigation into the effect of lingcod harvest on the long‐term outcomes of yelloweye, we found that when lingcod function as a generalist predator (γ=0.001), increased fishing pressure on lingcod, and therefore increased bycatch mortality of yelloweye, led to a linear reduction in yelloweye long‐term spawning biomass, stability, and age structure (Figure 5). Age structure experienced the greatest relative change as *F* increased, where the percent of the population in the plus group decreased by 40% when lingcod were fished at their management target ($F_{40}$) compared to when lingcod were not harvested (F=0; Figure 5c). Spawning biomass dropped by roughly 28% and stability by 10% (Figure 5a,b). This generalist scenario most closely resembles the true interaction shared by lingcod and yelloweye, which suggests that the negative impact of bycatch mortality outweighs the potential benefits of reduced predation pressure on the long‐term outcomes of yelloweye under the bycatch rate that we tested. However, while the long‐term spawning biomass of yelloweye decreased with lingcod harvest, it remained above the limit at which populations are declared overfished and fall under a rebuilding plan (typically 25% of unfished spawning biomass) across the range of lingcod harvest scenarios explored. These differences relative to the no fishing scenario remained consistent throughout recovery, indicating that the transient dynamics of yelloweye spawning biomass and age structure follow similar patterns to steady‐state outcomes across fishery scenarios (Appendix S1: Figure S2).

When we model lingcod as a specialist predator (γ=0.05), lingcod harvest results in slightly higher outcomes for the spawning biomass and stability of yelloweye than when we model lingcod as a generalist. Under specialist predation, spawning biomass of yelloweye at steady state was reduced by 17% when lingcod were fished at their management target ($F_{40}$) compared to when lingcod were not harvested (Figure 5a). Stability increased by 4%, which suggests that the reduction in predation pressure because of lingcod harvest can stabilize yelloweye populations in the long term given strong specialization by lingcod (Figure 5b). However, this positive outcome becomes negative under higher rates of bycatch mortality (Appendix S1: Figure S3). Lingcod harvest has the same influence on age structure regardless of prey specialization (Figure 5c) likely because older and larger yelloweye experience very little predation by lingcod due to gape limitation and are consequently more affected by bycatch mortality from lingcod harvest. Therefore, as prey specialization increases, the negative effect of lingcod harvest on spawning biomass and stability weakens, with an indication of possible (but slight) benefits to the long‐term stability of yelloweye.

**FIGURE 6:**
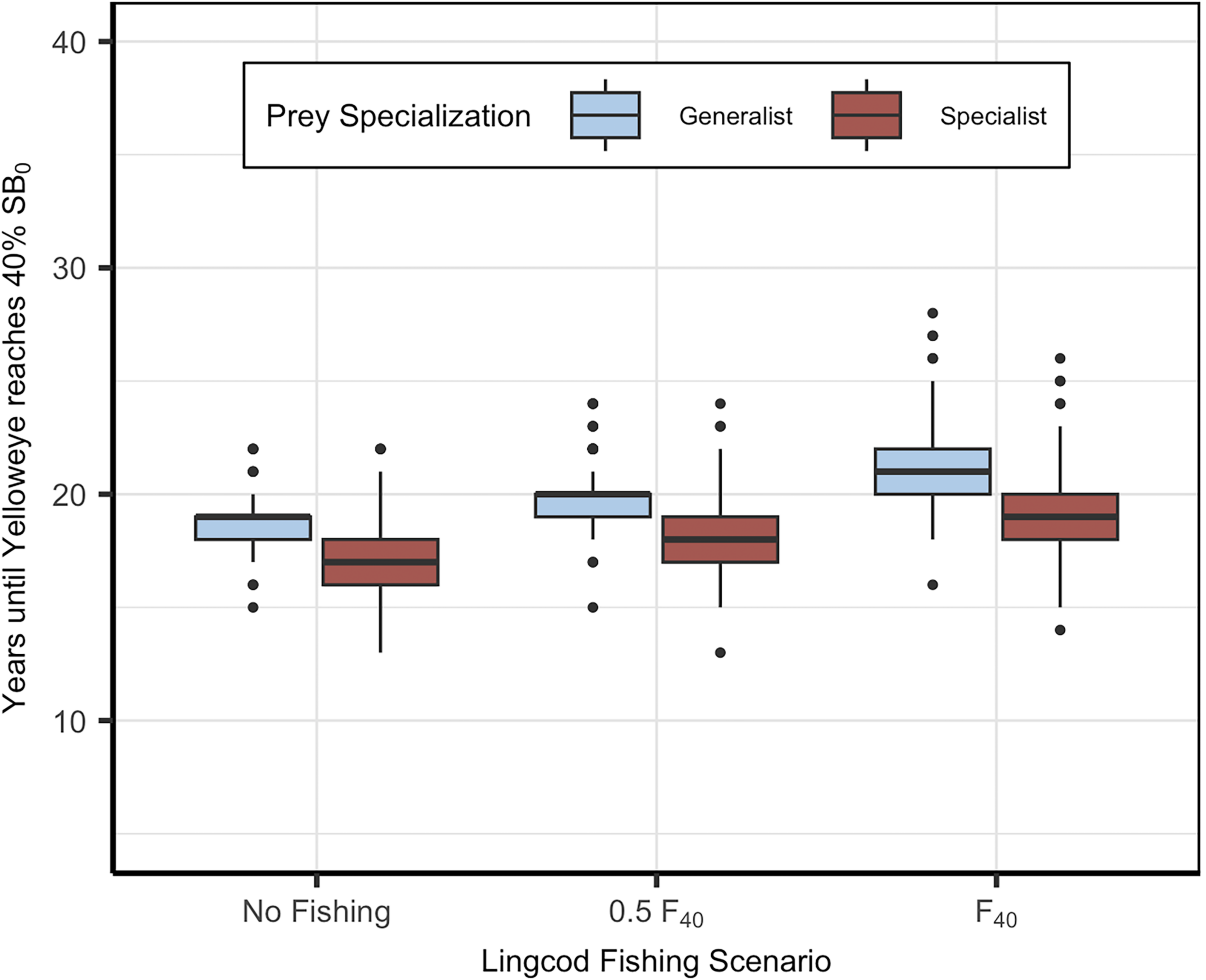
Rebuilding time of yelloweye rockfish across three lingcod fishing scenarios: no fishing ($\frac{F}{F_{40}}=0$), moderate fishing ($\frac{F}{F_{40}}=0.5$), and target fishing ($\frac{F}{F_{40}}=1$). We show rebuilding outcomes when lingcod are generalist predators (γ = 0.001; blue) and specialist predators (γ = 0.05; red). Variability in rebuilding time is driven by stochastic recruitment across 150 simulations.

The mean and variability in rebuilding time both depend on prey specialization and lingcod harvest. Under both prey specialization scenarios, mean rebuilding time increased with lingcod harvest (Figure 6) because yelloweye are subjected to greater bycatch mortality which slows their recovery. Mean rebuilding time increased by roughly 11% when lingcod were fished at $F_{40}$ compared to when lingcod were not harvested at all. Mean rebuilding time was consistently 10% faster when lingcod specialized on yelloweye compared to when lingcod acted as a generalist across all lingcod harvest scenarios. Variability in rebuilding time differed between prey specialization scenarios, ranging from 10% to 24% greater variability when lingcod were specialists compared to generalists. This difference in variability between specialist and generalist predators decreased with increasing lingcod harvest, such that lingcod harvest had a greater impact on variability when lingcod were generalists compared to specialists, exhibiting a 54% and 34% increase in variability, respectively, between a scenario where lingcod are not harvested and when lingcod are harvested at $F_{40}$. Rebuilding time and uncertainty were sensitive to the assumption of bycatch mortality for both prey specialization scenarios, with longer rebuilding times and more variability associated with higher bycatch mortality (Appendix S1: Figure S4).

## DISCUSSION

We found that, for our yelloweye rockfish case study, the positive effect of reduced predation pressure on a rebuilding prey through predator harvest can be outweighed by the negative effect of bycatch mortality on the long‐term and transient outcomes of rebuilding prey. The long‐term spawning biomass of our rebuilding prey, yelloweye, remained above our specified recovery benchmark (40% of its unfished spawning biomass) across all predator harvest scenarios, suggesting that they can rebuild given adequate time. Predator harvest led to small increases in rebuilding time, suggesting that bycatch mortality influences transient recovery, the extent of which intensified with increasingly indiscriminate fishing gear (*b* = 0.1; Appendix S1: Figure S3). Variability in rebuilding time increased with predator harvest. This is likely because fishing‐related mortality can reduce stability of prey directly through bycatch and indirectly through changes in the stability of harvested predators affecting their prey through consumption (Hsieh et al., 2006).

Prey specialization reduced the negative effect of predator harvest on the long‐term spawning biomass and stability of prey and reduced the time it took for prey to rebuild. When the trophic dependency of a predator on a prey increases (i.e., increased prey specialization), the predator has a stronger impact per capita on the spawning biomass and stability of prey through consumption. Therefore, the removal of a specialist predator can reduce the predation mortality experienced by prey more compared to the removal of a generalist predator. This can allow prey to rebuild more quickly and achieve greater long‐term spawning biomass. Harvesting a specialist predator improved the stability of prey by reducing the variability in predation mortality associated with natural and fishing‐related changes in predator abundance.

### Comparison to existing theories

These findings contribute to our growing understanding of ecological processes that govern community recovery. Initial theoretical work revealed the importance of timing as a factor governing the efficiency of community recovery. Simultaneous recovery, where predator and prey are protected at the same time, was the most efficient order of recovery for simple predator–prey systems (Samhouri et al., 2017), but considering more complex ecological processes highlights scenarios where alternative orders of recovery might prevail. For example, in a tri‐trophic and size‐structured kelp forest community, protecting predators first supported the most efficient path of recovery (Dunn et al., 2021) likely because kelp forest communities depend on the top‐down control of grazers (Hamilton & Caselle, 2015; Tegner & Dayton, 2000). Including size‐structured predation increased recovery time because of gape limitation while external recruitment decreased recovery time because of increased productivity (Dunn et al., 2021). In our study, we included prey specialization and bycatch mortality as additional processes that might affect recovery and found that increasing prey specialization can decrease the recovery time of prey regardless of whether the predator and prey were protected simultaneously (“No Fishing” in Figure 6) or prey were protected first (*F*_40_ in Figure 6). Across both prey specialization scenarios, simultaneous protection of predator and prey resulted in the most effective recovery for prey, suggesting that bycatch mortality has strong consequences to recovery in this system.

We found that prey rebuilt more quickly when their predators were specialists compared to generalists. A similar study, however, found that increasing prey specialization prolonged rebuilding time for a similar long‐lived and overfished rockfish species (Harvey et al., 2008). Differences in our estimates of rebuilding time might stem from differences in how we structured size‐specific predation in our models, as well as differences in how we calculated rebuilding time. Harvey et al. (2008) modeled predation on only age‐0 recruits of rockfish, whereas we allowed predation across a wider range of age classes. Such targeted predation pressure on immature individuals removes their ability to contribute to the population through reproduction, thereby reducing productivity and slowing recovery. Alternatively, rebuilding time can be largely affected by how we define the unfished state in which a population is rebuilding toward. In our study, total mortality included a fixed baseline natural mortality across all prey specialization scenarios, plus predation mortality that varied between those scenarios. As such, unfished spawning biomass depended on prey specialization; prey rebuilt to a smaller unfished spawning biomass under specialist predation compared to generalist predation, likely contributing to the faster rebuilding timelines observed in our study.

### Management insights

Rebuilding plans are traditionally developed and implemented on a species‐by‐species basis, but the management strategies used to support them, such as spatial closures, can affect or be affected by the biological community in which the rebuilding species exists (Baskett et al., 2007; Marshall et al., 2016). This mismatch in conception inherently sets up conflicts among single‐species management strategies, causing concern for fisheries that target productive but inaccessible species like lingcod or that simultaneously target multiple species that all sustain different levels of fishing pressure (Fogarty, 2014; Hilborn et al., 2012; Moore et al., 2021). Moreover, species interactions that are unaccounted for can lead to unexpected outcomes within marine protected areas (Baskett et al., 2007), even though marine protected areas are often expected to support the recovery of species under rebuilding plans. Theoretical research regarding the role of species interactions in recovery is growing (Dunn et al., 2021; Samhouri et al., 2017), and we build upon this work to consider species interactions in the context of recovery strategies commonly used in single‐species rebuilding plans.

Consistent across studies, predator harvest within RCAs increases the rebuilding time of recovering species (Harvey et al., 2008). A critical component of rebuilding plans in the United States are their strict rebuilding timelines, and prolonging rebuilding time may violate rebuilding mandates despite its potential benefits to other recovering populations and to coastal communities. As rebuilding plans are primarily designed to restore overfished stocks, their rigid conservation strategies can harm fishing communities. For example, following the establishment of rebuilding plans for overfished groundfish along the U.S. West Coast, sharp reductions in catch levels resulted in job losses, reduced income, and broad economic hardships, which disrupted the livelihoods of thousands of people (Conway & Shaw, 2008). Similar socioeconomic impacts following sudden reductions in catch (e.g., fishery moratoria) have been documented in other lucrative fisheries, including Dungeness crab (Moore et al., 2020) and salmon (Richerson & Holland, 2017), making it important to explore avenues that minimize such consequences.

Here, we demonstrate the outcomes of considering biological interactions within rebuilding plans as one potential avenue that can be applied to a system characterized by a productive abundant predator and a slowly recovering prey. While we did not find the opportunity for a win‐win outcome, where lingcod harvest increases both economic opportunities and yelloweye recovery via predation release, our results indicate that addressing the challenge of bycatch can help reduce the socioeconomic cost associated with unrealized harvest. Gear designed to minimize bycatch of nontarget species is one way to help offset the direct negative effects of bycatch while still supporting the harvest of healthier stocks (King et al., 2004; Starr et al., 2016). For example, in 2003, the flatfish trawl fishery along the U.S. West Coast experimented with selective fishing gear that reduced bycatch of vulnerable rockfish species while fishing on productive grounds within the RCA (King et al., 2004; Parker et al., 2004). Collaborative research with local and indigenous fishing communities have successfully modified gear to catch abundant species while limiting bycatch of rebuilding species like yelloweye rockfish (Starr et al., 2016; Stewart et al., 2021). Moreover, further development of statistical models that estimate the species distributions at fine spatial resolution can identify locations where spatial overlap between target and nontarget species are at a minimum to reduce instances of bycatch (Stock et al., 2020).

### Model caveats

As prey specialization increased, the total non‐fishing mortality (predation plus other natural mortality) experienced by yelloweye also increased which can affect recovery dynamics. In our model, we used an estimate of other natural mortality $M^{Y}$ from a single‐species stock assessment model (Gertseva & Cope, 2017) which remained fixed across prey specialization scenarios. While this value from the literature is theoretically inclusive of lingcod predation and potentially inflates the total natural mortality in our model, we note that this is a stylized model. Following a sensitivity analysis on natural mortality, we found that decreasing natural mortality increased the spawning biomass of yelloweye rockfish at steady state (Appendix S1: Figure S5) which can affect our comparison of recovery time across prey specialization scenarios. This modeling decision likely influenced our finding that prey recovery was quicker when predators were specialists compared to generalists. However, it is important to note that natural mortality was a key source of uncertainty in the single‐species stock assessment model (Gertseva & Cope, 2017) and was fixed based on the maximum observed age (i.e., unable to be estimated). For U.S. West Coast groundfish stock assessments that do estimate natural mortality, the rate based on the maximum age tends to be lower than the estimated value (e.g., Johnson et al., 2023).

For consistency in model structure across scenarios, we assumed that changes in yelloweye abundance did not affect lingcod dynamics across all prey specialization scenarios (i.e., we modeled a one‐way interaction). This assumption is most appropriate for lingcod because they are generalist predators and can find alternative resources when yelloweye are absent. However, specialist predators are more likely to be affected by changes in their prey and, as such, we would expect a two‐way interaction as prey specialization increases. We might expect that accounting for this two‐way interaction at higher prey specialization scenarios would result in slower rebuilding times because lingcod productivity would increase as yelloweye recover, potentially increasing consumption of yelloweye.

While we analyzed a wide range of direct fishing mortality, we did not explore a similar range in bycatch mortality experienced by yelloweye which can affect estimates of their dynamics at steady state and transient recovery. We explored one additional scenario where bycatch was doubled, which resulted in sharper declines in the steady‐state dynamics of yelloweye with increased lingcod harvest, as well as a stronger influence of fishing pressure on rebuilding time, greater uncertainty in rebuilding time, and greater differences in rebuilding time between generalists and specialists (Appendix S1: Figures S3 and S4). In reality, bycatch is not often perfectly coupled with direct fishing pressure and can instead depend on factors such as the level of spatial overlap between species, the type of fishing gear, and individual vessel behavior (Hatch et al., 2023; Roberson & Wilcox, 2025). We find that fishing‐related mortality has a greater influence on yelloweye dynamics compared to predation mortality, so an accurate representation of fishery dynamics, with a particular focus on bycatch mortality, will be essential in determining whether a recovery approach that considers species interactions will lead to an expected outcome.

In our model, we focus on the interaction of two species where one consumes the other. Yet predators and prey exist within a larger network of species that interact in many ways. To account for this complexity, food web models have been developed to capture both direct and indirect interactions (Koehn et al., 2016; Marshall et al., 2017) and will likely give insight into a wider and more accurate range of recovery dynamics. However, the network surrounding yelloweye and lingcod might be incredibly complex given that both species are generalist predators and are likely to interact with many different species. Such complexity can make it challenging to disentangle the factors influencing the recovery dynamics of yelloweye rockfish and introduces many additional parameters that lack data to support their values. Nevertheless, understanding the outcomes of recovery in such complex networks can support the development of management strategies that provide both conservation and socioeconomic benefits.

Finally, we find that prey specialization, recruitment variability, and recruitment autocorrelation play a central role in the population dynamics and rebuilding of yelloweye rockfish. Species‐specific values for these components of our model were highly uncertain, except for recruitment variability, and could benefit from further resolution through empirical studies. Here, we account for these uncertainties by testing a range of values through local sensitivity analyses. We found that increases in the variability and autocorrelation of yelloweye recruitment were associated with increases in the fraction of older fish (e.g., age structure) but reductions in spawning biomass and stability (Appendix S1: Figure S6). Despite uncertainty in the true value of these parameters and others, this work primarily contributes to our theoretical understanding of recovery when biological and fishery interactions are considered rather than a tactical application to the Yelloweye rockfish rebuilding plan.

## CONCLUSION

Biological interactions remain a fundamental challenge in fisheries management. The importance of biological interactions and food webs has been recognized and explored in ecological theory and fisheries research for many decades (Fogarty, 2014; May et al., 1979; Murawski, 1991; Paine, 1980; Wootton, 1994). However, its implementation into fisheries management strategies has not sustained the same momentum despite long‐standing calls for more holistic and ecosystem‐based management approaches (Botsford et al., 1997; Pikitch et al., 2004; Trochta et al., 2018). In this paper, we consider species interactions in the context of rebuilding plans and highlight the importance of prey specialization and bycatch mortality on the recovery of a species consumed by an abundant, harvested predator. Expanding this model to simulate theoretical predator–prey systems with different life history traits will provide insight into which combinations of traits are more sensitive to prey specialization during prey recovery and, more broadly, in which systems predator harvest can lead to favorable outcomes in prey recovery. Given the importance of prey specialization, empirical diet studies that utilize molecular tools to identify prey at the species level might improve the application of this model to real systems such as yelloweye rockfish and lingcod.

## AUTHOR CONTRIBUTIONS

Andrea N. Odell and Kiva L. Oken conceived the study. All coauthors contributed to the mathematical development of the model. Andrea N. Odell ran model simulations and analyses. Andrea N. Odell led writing with comments and multiple rounds of feedback from all coauthors.

## CONFLICT OF INTEREST STATEMENT

The authors have no conflicts of interest to declare.

## Supporting information


Appendix S1.

## Data Availability

Code (Odell, 2026) to generate the model and run the analyses is available in Zenodo at https://doi.org/10.5281/zenodo.19407626.
