# Supplementary material for: The outcomes of integrating biological interactions into rebuilding plans depend on prey specialization
Source: Ecol Appl. 2026 Jun 5;36(4):e70269. doi: 10.1002/eap.70269 (PMC13238822; doi:10.1002/eap.70269)
Supplement: Supplementary file 1 — Appendix S1. [file EAP-36-e70269-s001.pdf]

Appendix S1

The outcomes of integrating biological interactions into rebuilding plans depend on prey specialization

Andrea N. Odell, Kiva L. Oken, Marissa L. Baskett

Ecological Applications

Section S1

Calculating the $\mathbf{a_{i,j,s}}$ matrix and q

We closely followed methods from Oken and Essington (2016) to calculate a matrix of size-selective rates of per capita consumption of age *i* yelloweye by an age *j* and sex *s* lingcod, which we briefly describe here. We combined three pieces of information: total annual consumption by lingcod age j and sex s, lingcod age j and sex s preferences for different prey sizes, and the proportion of consumed rockfish biomass that is yelloweye specifically (which controls prey specialization; γ).

Total annual consumption by lingcod age j and sex s

Using lingcod consumption parameters a_s^c and b_s^c (Table 1; Beaudreau and Essington, 2009), we empirically derived the total daily consumption (g) by lingcod and converted this value into a vector of annual per capita consumption (kg) of an age *j* and sex *s* lingcod ($C_{j,s}^L$) following $C_{j,s}^L = \frac{365}{1000} a_s^c {W_{j,s}^L}^{b_s^c}$, where $W_{j,s}^L$ is the vector of weight-at-age for each lingcod sex. Because the consumption parameters were obtained using samples from the Puget Sound in Washington, we corrected these values using the studies' reported Q_{10} temperature coefficient (Q_{10} = 1.9) for the lower temperatures found on the continental shelf when compared to Puget Sound (6 vs. 9°C).

Lingcod age j and sex s preferences for different prey sizes

We assumed that an age *j* and sex *s* lingcod's preference for different prey sizes follows a gamma distribution, gamma(α_j, β_j), where β is the scale parameter. Based on previous research on lingcod diets, this gamma distribution uses number of prey consumed and measures prey sizes in standard length to represent lingcod size preferences. We assumed the 5th and 95th quantiles of the gamma distribution increased linearly with lingcod sizes, which represents the shift in the size spectra of prey items observed in an age *j* lingcod's diet. This can be represented as

gamma($\alpha, \beta S_j^L$), where the parameters are constant across lingcod size and S_j^L is the size of an age j lingcod.

We used this distribution to quantify a lingcod's relative preference for an age i yelloweye. We defined an interval of prey sizes for an age i yelloweye where breakpoints were halfway between the length-at-age of consecutive age classes. We integrated the gamma distribution over each size-at-age bin and normalized these values so that they summed to one. Because these values are represented as number of prey consumed, we converted the size spectra to weight by multiplying the relative preference in each length bin by the associated weight of a yelloweye of that length, and then re-normalized the vector. This resulted in a vector of diet weighting factors where each yelloweye age class had one value, and this process was repeated for each lingcod age and sex. These vectors were used to populate a matrix D, of $r \times 2l$ where r is the number of yelloweye age classes and l is the number of lingcod age classes. Each of the columns in this matrix represents a vector of an age j sex s lingcod's preference for each yelloweye age class, and sums to one.

Calculating yelloweye instantaneous consumption rates

We assumed yelloweye rockfish was a constant fraction of an age j sex s lingcod's diet, γ. We multiplied $C_{j,s}^L$ and γ to obtain total annual consumption of yelloweye by an age j sex s lingcod. We distributed total annual consumption of yelloweye across yelloweye age classes by multiplying $\gamma C_{j,s}^L$ by the preference matrix D. This created a matrix, $Q_{i,j,s}$, of total annual consumption (kg) of an age i yelloweye by each age j sex s lingcod. We converted this matrix from weight (kg) to numbers because our population model tracks numbers at age. We then converted this matrix from annual rates to instantaneous rates using $a_{i,j,s} = -\ln\left(1 - \frac{Q_{i,j,s}}{N_i^Y}\right)$, where N_i^Y is the number of age i yelloweye.

Calculating alternate prey availability

We calculated the alternate prey availability, q, following the function $q = \frac{\sum_j \sum_s \frac{Q_{i,j,s}}{N_i^Y}}{\gamma}$, where we summed the matrix of total annual consumption (in numbers; $\frac{Q_{i,j,s}}{N_i^Y}$) of age i yelloweye across all age j sex s lingcod and divided by the proportion of lingcod diet that is specifically yelloweye, γ.

Please refer to Oken and Essington (2016) supplementary materials for further details and equations.

Tables

Table S1. Distribution of parameter values randomly selected from for the global sensitivity analysis.

Symbol	Description	Distribution
δ	Handling time	U(0.1, 1)
Y_{95}	Slope of ninety-fifth quantize of diet size spectra	U(0.27, 0.33)
ρ^L	Recruitment autocorrelation- lingcod	U(0.1, 0.7)
ρ^Y	Recruitment autocorrelation- yelloweye	U(0.1, 0.7)
σ	Recruitment variability	U(0.3, 0.7)
γ	Prey specialization	U(0.0001, 0.05)

Figures

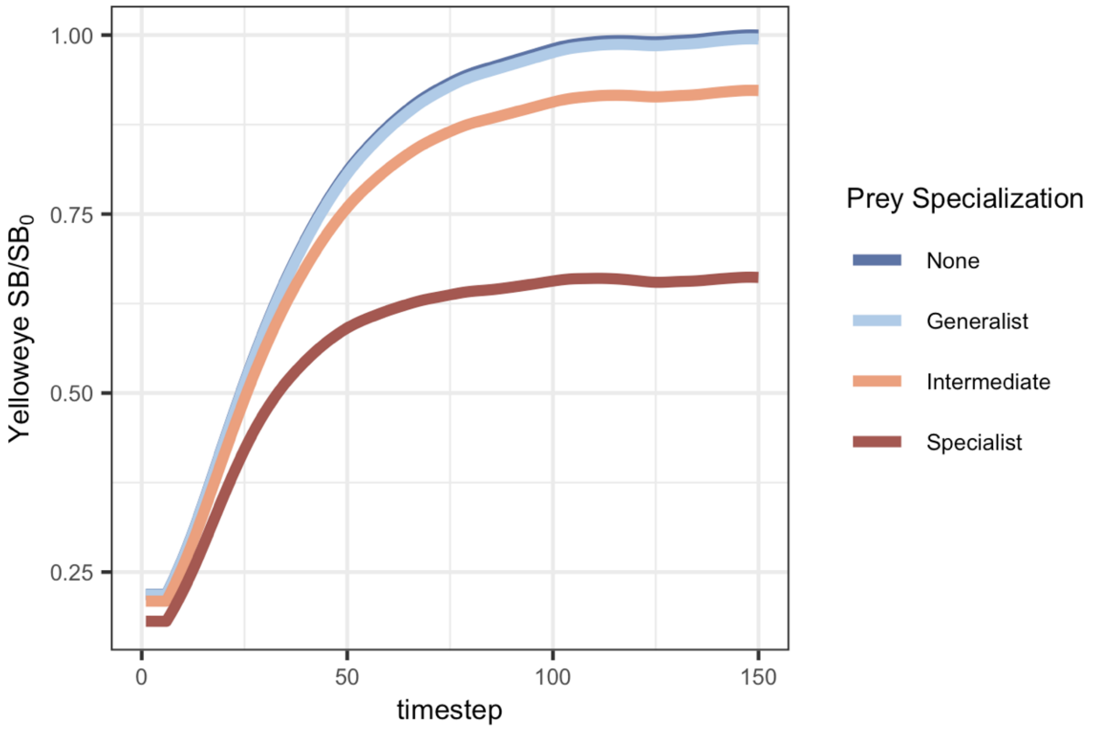

Figure S1. Transient dynamics of yelloweye rockfish spawning biomass relative to the maximum biomass in the “none” scenario across all prey specialization scenarios (in color). Each timeseries was calculated by taking the average population size at timestep *t* across all simulations for each prey specialization scenario. Timestep is in years.

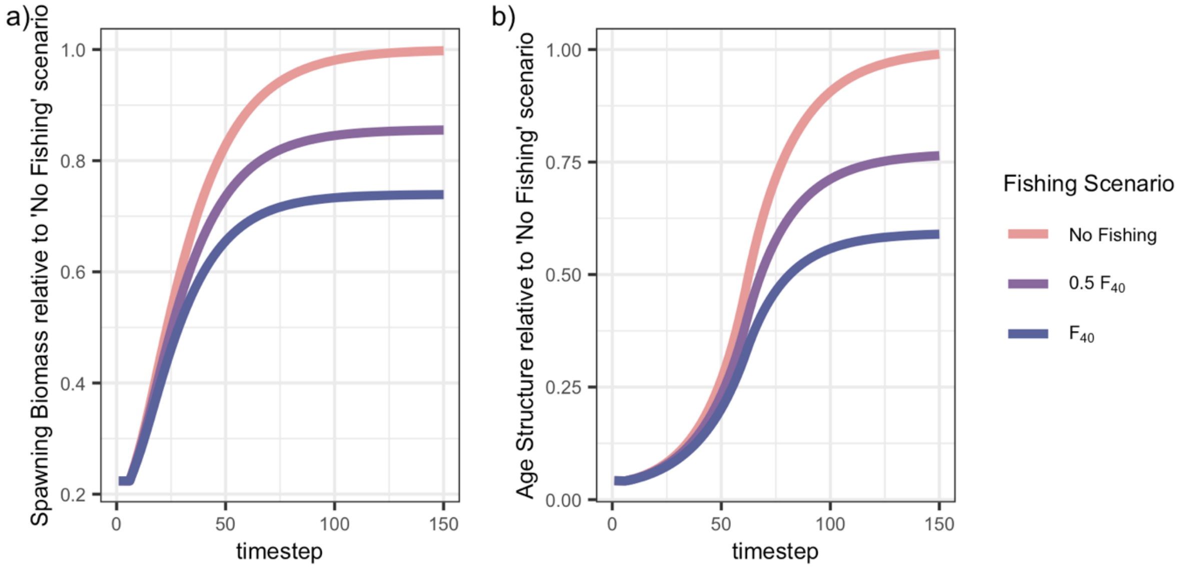

Figure S2. Transient dynamics of yelloweye rockfish a) spawning biomass and b) age structure relative to the terminal value of the “none” scenario timeseries across three lingcod fishing

scenarios (in color): No fishing ($\frac{F}{F_{40}} = 0$), moderate fishing ($\frac{F}{F_{40}} = 0.5$), and target fishing ($\frac{F}{F_{40}} = 1$). Each timeseries was estimated using a deterministic model where each timestep is one year and lingcod were modeled as generalist predators.

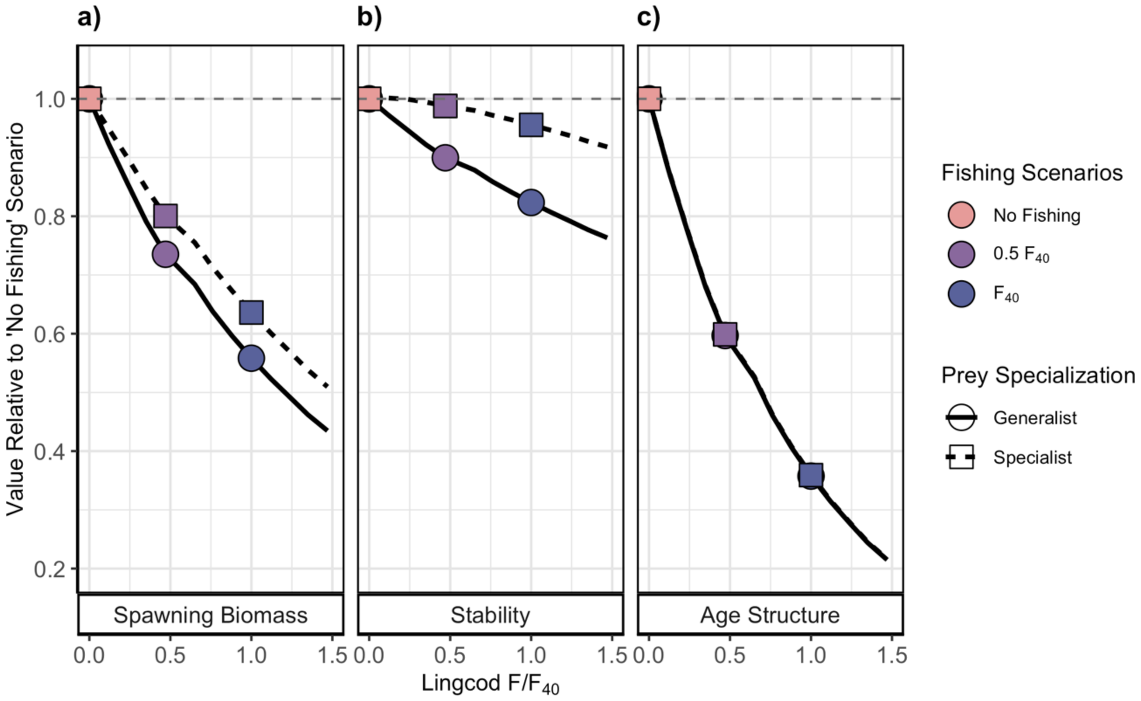

Figure S3. Steady state outcomes of yelloweye rockfish a) spawning biomass, b) stability, and c) age structure experiencing double the bycatch (b = 0.1) related to increasing lingcod fishing pressure F relative to a fishing rate that achieves 40% of the unfished biomass for lingcod F_{40} for lingcod as a generalist predator (solid line and points) and a specialist predator (dashed line and open points).We visualize outcomes in relation to the no fishing scenario ($\frac{F}{F_{40}} = 0$) scenario, where the values are represented as the proportion relative to the outcome expected if a lingcod fishery did not operate. Points indicate fishing scenarios ($\frac{F}{F_{40}} = 0$, $\frac{F}{F_{40}} = 0.5$, and $\frac{F}{F_{40}} = 1$) explored for rebuilding time.

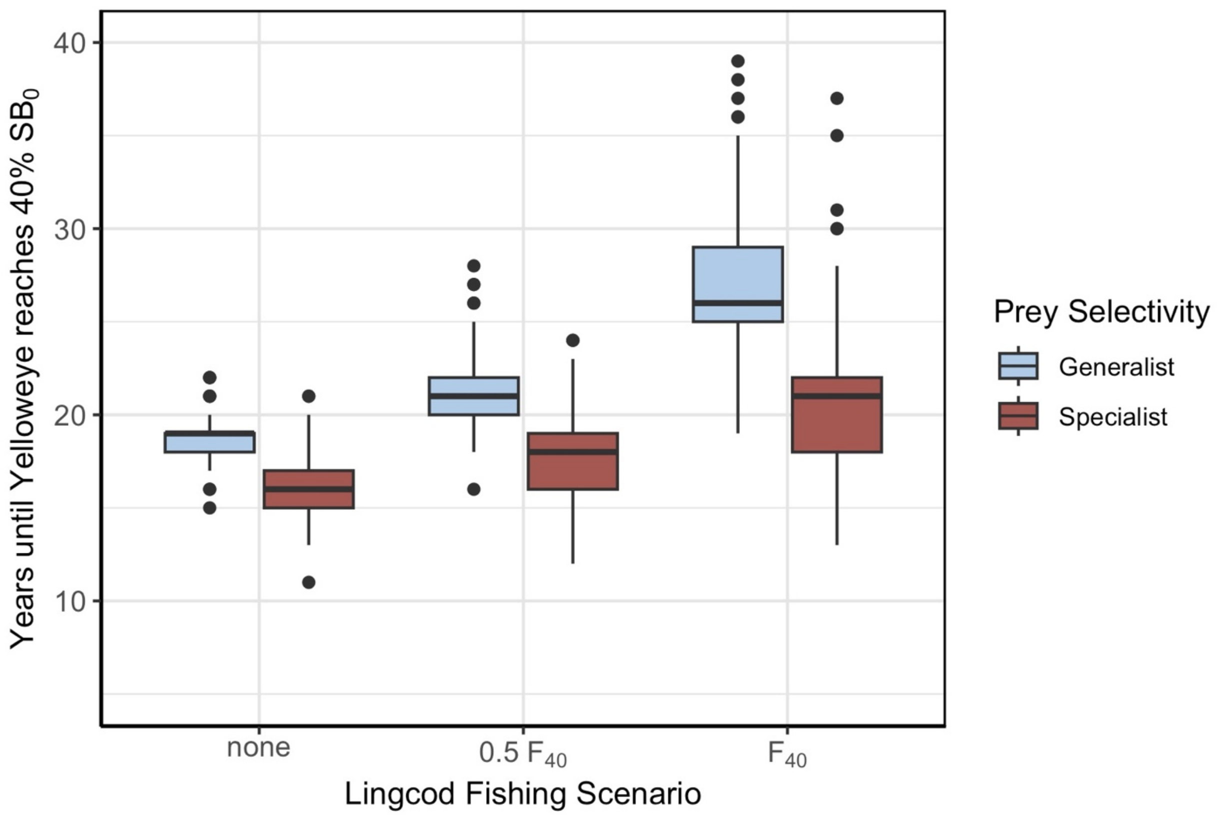

Figure S4: Rebuilding time of yelloweye rockfish experiencing double the bycatch (b = 0.1) across three lingcod fishing scenarios: No fishing ($\frac{F}{F_{40}} = 0$), moderate fishing ($\frac{F}{F_{40}} = 0.5$), and target fishing ($\frac{F}{F_{40}} = 1$). We show rebuilding outcomes when lingcod are generalist predators (γ = 0.001; blue) and specialist predators (γ = 0.05; red). Variability in rebuilding time is driven by stochastic recruitment across 150 simulations.

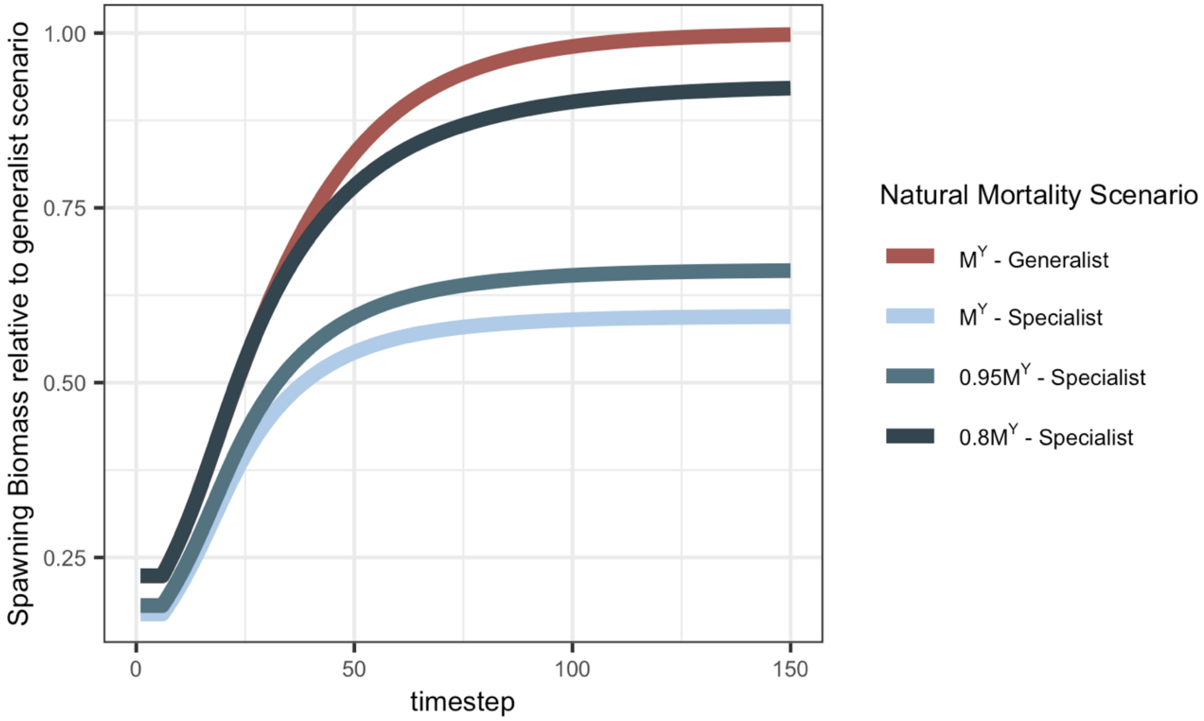

Figure S5. Sensitivity analysis for natural mortality demonstrated as a timeseries of yelloweye spawning biomass for generalist and specialist predation scenario at base parameterization (e.g., $M^Y = 0.044$), and two additional natural mortality parameterizations for the specialist predation scenario: $0.8M^Y$ and $0.95M^Y$. Reduced natural mortality values for the specialist predation scenario indicates the potential recovery outcomes when standardizing total mortality across prey specialization scenarios.

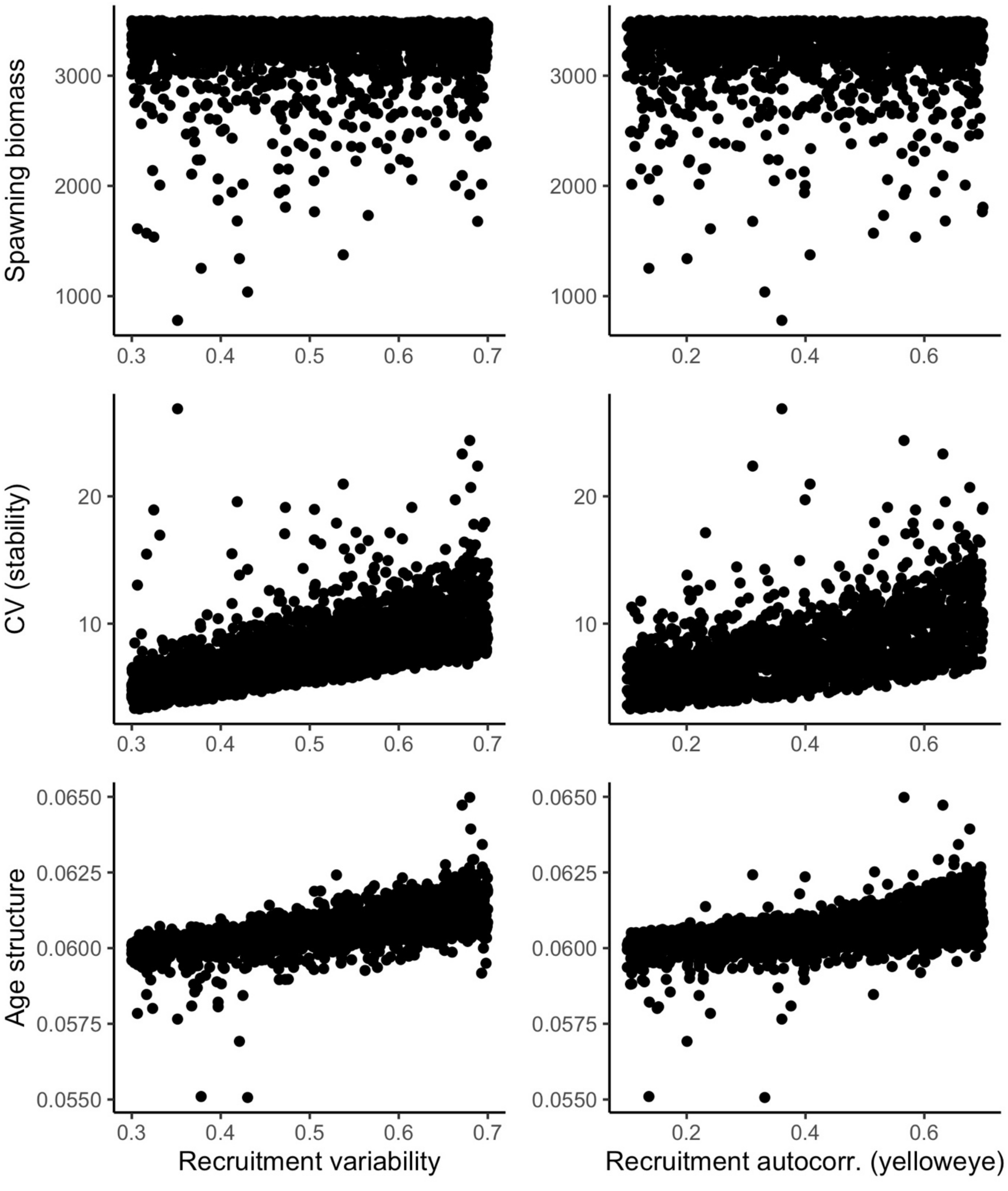

Figure S6. Outputs of global sensitivity analysis showing relationship between steady-state outcomes (spawning biomass, stability, and age structure) and variability in recruitment (left column) and recruitment autocorrelation (right column) in yelloweye rockfish.

References

Oken, K.L., Essington, T.E., 2016. Evaluating the effect of a selective piscivore fishery on rockfish recovery within marine protected areas. ICES J Mar Sci 73, 2267–2277.
